# Xpert MTB/RIF Ultra-resistant and MTBDR*plus-*susceptible rifampicin results in people with tuberculosis: utility of FluoroType MTBDR and deep sequencing

**DOI:** 10.1128/aac.01671-24

**Published:** 2025-02-07

**Authors:** Yonas Ghebrekristos, Aysha Ahmed, Natalie Beylis, Sarishna Singh, Christoffel Opperman, Fahd Naufal, Megan Folkerts, David Engelthaler, Erick Auma, Rouxjeane Venter, Ghowa Booley, John Metcalfe, Robin Warren, Grant Theron

**Affiliations:** 1 DSI-NRF Centre of Excellence for Biomedical Tuberculosis Research, and SAMRC Centre for Tuberculosis Research, Division of Molecular Biology and Human Genetics, Faculty of Medicine and Health Sciences, Stellenbosch University 26697 , Cape Town, South Africa; 2 National Health Laboratory Service, Greenpoint Tuberculosis Laboratory, Cape Town, South Africa; 3 Division of Medical Microbiology, Department of Pathology, University of Cape Town 37716 , Cape Town, South Africa; 4 University of California San Francisco 8785 , San Francisco, California, USA; 5 Translational Genomics Research Institute 10897 , Phoenix, Arizona, USA; Bill & Melinda Gates Medical Research Institute, Cambridge, Massachusetts, USA

**Keywords:** tuberculosis, diagnosis, rifampicin susceptibility, heteroresistance

## Abstract

Xpert MTB/RIF Ultra (Ultra)-detected rifampicin-resistant tuberculosis (TB) is often programmatically confirmed using MTBDR*plus*. There are limited data on discordant results, including when re-tested using newer methods, like FluoroType MTBDR (FT-MTBDR) and targeted deep sequencing. MTBDR*plus* rifampicin-susceptible isolates from people with Ultra rifampicin-resistant sputum were identified from a South African programmatic laboratory. FT-MTBDR and single molecule-overlapping reads (SMOR; *rpoB*, *inhA*, *katG*) on isolate DNA were done (SMOR was used as a reference standard). Between 1 April 2021 and 30 September 2022, 8% (109/1347) of Ultra rifampicin-resistant specimens were MTBDR*plus*-susceptible. Of 89% (97/109) isolates with a sequenceable *rpoB*, SMOR resolved most in favor of Ultra (79% [77/97]). Sputum with lower mycobacterial load was associated with Ultra false-positive resistance (46% [11/24] of “very low” Ultra had false resistance vs 12% [9/73; *P* = 0.0004] of ≥“low”), as were Ultra heteroresistance calls (all wild-type probes, ≥1 mutant probe) (62% [23/37 vs 25% 15/60] for Ultra without heteroresistance calls; *P* = 0.0003). Of the 91% (88/97) of isolates successfully tested by FT-MTBDR, 55% (48/88) were FT-MTBDR rifampicin-resistant and 45% (40/88) susceptible, translating to 69% (47/68) sensitivity and 95% (19/20) specificity. In the 91% (99/109) of isolates with *inhA* and *katG* sequenced, 62% (61/99) were SMOR isoniazid-susceptible. When Ultra and MTBDR*plus* rifampicin results are discordant, Ultra is more likely to be correct, and FT-MTBDR agrees more with Ultra than MTBDR*plus*; however, lower load and the Ultra heteroresistance probe pattern were risk factors for Ultra false rifampicin-resistant results. Most people with Ultra–MTBDR*plus* discordant resistance results were isoniazid-susceptible. These data have implications for drug-resistant TB diagnosis.

## INTRODUCTION

Xpert MTB/RIF Ultra (Ultra; Cepheid, Sunnyvale, USA) is a widely used test for diagnosing tuberculosis (TB) and detecting rifampicin resistance. Endorsed by the WHO, Ultra has been an essential screening tool in high-incidence countries, such as South Africa, where it has been used routinely since 2011. At the time of this study, in the South African TB Control program, if Ultra detected *Mycobacterium tuberculosis* complex (MTBC) and rifampicin resistance, a second specimen is typically cultured. The resulting MTBC isolate can be tested with GenoType MTBDR*plus* VER 2.0 (MTBDR*plus,* Bruker-Hain Lifescience, Nehren, Germany), which confirms rifampicin resistance and can additionally detect isoniazid resistance. However, discrepancies between results obtained directly from the patient specimen and those from cultured isolates or other molecular assays can occur, complicating both reporting and clinical management. This discordance, which could be due to heteroresistance, can lead to poor outcomes (1), patient distress, and significant financial burden due to delays and additional testing.

Although superseded in some settings by FluoroType MTBDR VER 2.0 (FT-MTBDR; Bruker-Hain Lifescience, Nehren, Germany) (2, 3), MTBDR*plus* is widely used for confirmatory drug susceptibility testing (DST). Both Ultra and MTBDR*plus* target the *rpoB* rifampicin-resistant determining region (RRDR) of MTBC. Reports from our high burden setting of South Africa highlight discordance in ~7% of Xpert MTB/RIF (Xpert; Ultra’s predecessor) resistant samples, which were MTBDR*plus* rifampicin-susceptible (4, 5). Sub-optimal Xpert readouts, particularly in the “very low” semi-quantification category, and probe delay have been linked to false rifampicin-resistant calls (6). However, this has not yet been studied in the context of Ultra. Aside from factors, like human error or cross-contamination, discordant results may also arise due to heteroresistance and culture bias, as Ultra is performed directly on specimens, while MTBDR*plus* is typically conducted on cultured isolates (7–9).

Two additional critical gaps exist. First, although Ultra itself does not directly report rifampicin heteroresistance, Ultra-reported probe melting temperatures have been suggested as a potential tool for inferring heteroresistance (if a specific probe has melting temperatures corresponding to both wild-type and mutant strains) (10). However, the diagnostic accuracy of such Ultra’s heteroresistance calls on clinical specimens has not been evaluated. Second, it is unclear whether the level of discordance between Ultra and FT-MTBDR, which utilizes LiquidArray technology to detect MTBC and mutations in *rpo*B, *inh*A, and *kat*G genes, is comparable to that observed with MTBDR*plus* (2).

We sought to address these knowledge gaps in individuals identified programmatically as having discordant rifampicin results (Ultra-resistant, MTBDR*plus*-susceptible). To ascertain true rifampicin susceptibility status, we employed targeted deep sequencing with single-molecule-overlapping reads (SMORs) as a reference standard (7, 9). SMOR detects resistant allele subpopulations at ≥0.1% in near real-time compared with whole genome sequencing, which reliably detects mutations at 5%–10%, depending on sequencing depth (11, 12). Additionally, we used FT-MTBDR as a comparator and analyzed isoniazid susceptibility using different tests as, in people with rifampicin-resistant TB, isoniazid may still have clinical utility. Our study also sought to identify test parameters associated with discordance, heteroresistance, and rifampicin mono-resistance.

## MATERIALS AND METHODS

### Study design and setting

The study was conducted from 1 April 2021 to 30 September 2022, using patient specimens and their corresponding isolates processed at the high-throughput National Health Laboratory Service (NHLS) Greenpoint TB Laboratory (Cape Town, South Africa; ~60,000 TB tests per month).

### Routine diagnostic algorithm

Following the diagnostic algorithm, healthcare workers collected two sputum samples an hour apart from individuals with presumptive TB not presently on TB treatment. Upon laboratory receipt, one specimen was arbitrarily selected for testing with Ultra and processed according to the manufacturer’s instructions (13). If Ultra detected MTBC and rifampicin resistance, the second specimen was processed for mycobacterial culture using the standard N-acteyl-L-cysteine-sodium hydroxide (NALC-NaOH) (1.25% final concentration) decontamination procedure and 0.5 mL inoculated into a *Mycobacterium* Growth Indicator Tube 960 (MGIT960; Becton Dickinson Diagnostic Systems, Sparks, USA) supplemented with polymyxin B (400 units/mL), amphotericin B (40 µg/mL), nalidixic acid (160 µg/mL), trimethoprim (40 µg/mL), and azlocillin (40 µg/mL) (PANTA, Becton Dickinson Diagnostic Systems) and incubated for ≤35 days. After a tube is automatically flagged growth-positive, Ziehl–Neelsen (ZN) microscopy was performed to detect acid-fast bacilli (AFB). If AFBs were observed, MTBDR*plus* was conducted on the MGIT culture according to the manufacturer’s protocol, using the GenoScan instrument with semi-automated reading and manual confirmation (14). The MGIT tubes were stored at room temperature for up to 7 days until DNA extraction for MTBDRplus, FT-MTBDR, and SMOR analysis. All assays were performed once, with no repeat testing.

### Discordant isolate selection and definition of Ultra heteroresistance results

**Fig 1:**
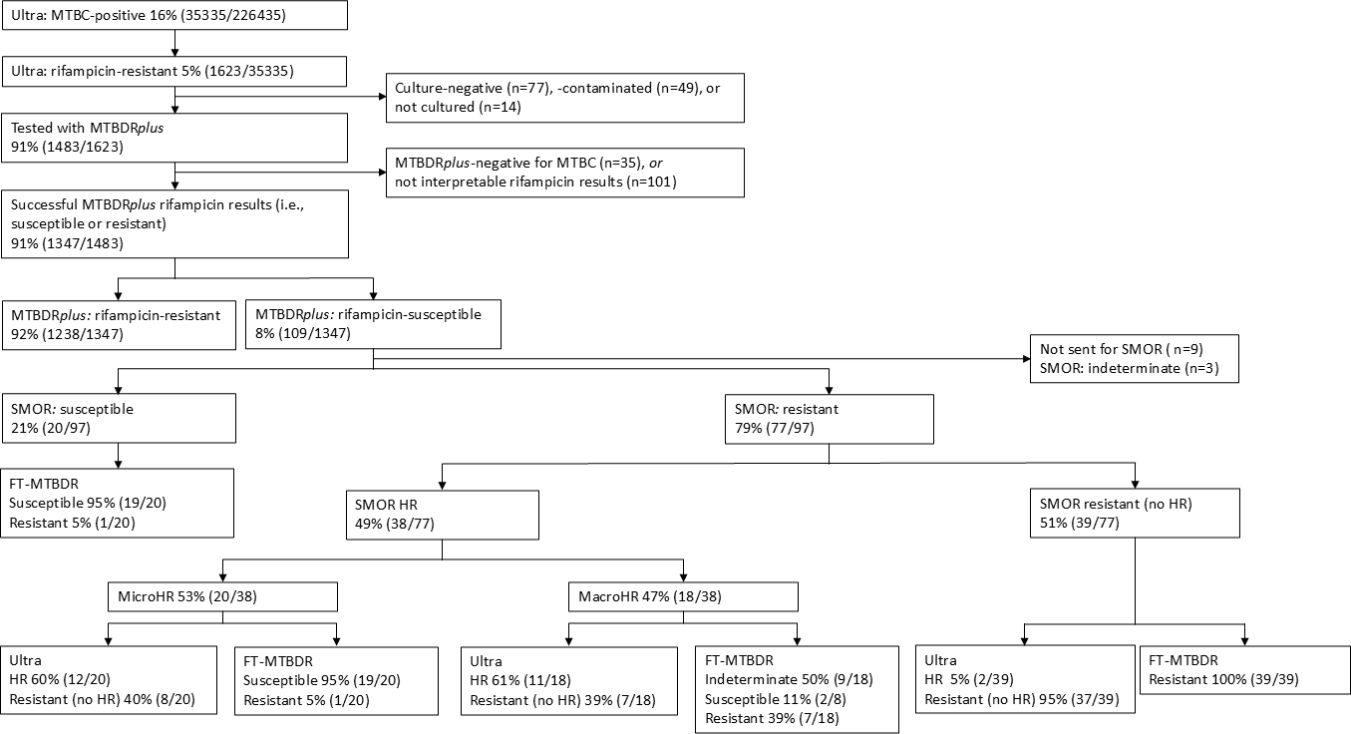
Study profile. We quantified discordant rifampicin susceptibility results (Ultra-resistant, MTBDR*plus*-susceptible) done at TB diagnosis on respiratory specimens over an 18-month period. The distribution of HR is shown, and most isolates that were confirmed by sequencing have RAVs missed by MTBDR*plus* but often detected by FT-MTBDR. Abbreviations: FT-MTBDR, FluoroType MTBDR; MicroHR, microheteroresistance; MacroHR, macroheteroresistance; MTBC, *Mycobacterium tuberculosis* complex; SMOR, single molecule-overlapping repeats; RAV, resistance-associated variant; TB, tuberculosis; Ultra, Xpert MTB/RIF Ultra.

**Fig 2:**
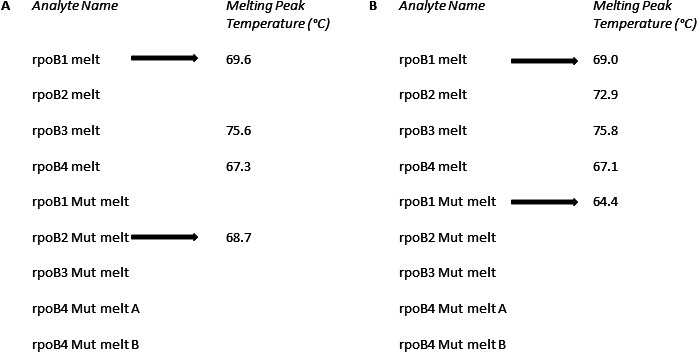
Examples of an Ultra report generated by the GeneXpert software showing melt peak temperatures for each amplicon. (**A**) A commonly seen rifampicin-resistant specimen with a variant in the rpoB2 region. There is no indication of HR because, for rpoB2, “melt” has no value but “Mut melt” does (black arrows). (**B**) In contrast, rpoB1 has both “melt” and “Mut melt” values, suggesting HR. Abbreviations: °C; degree Celsius; HR, heteroresistance; melt, wild-type melt peak temperature; Mut melt, mutant melt peak teak temperature; Ultra, Xpert MTB/RIF Ultra.

We selected MTBDR*plus* rifampicin-susceptible isolates from specimens collected concurrently with those tested by Ultra (Fig. 1). Patients were classified as Ultra heteroresistant based on the melting temperature curve peaks for each *rpoB* probe as previously described (10). Briefly, if each probe exhibited melting peaks corresponding to the wild-type temperature in addition to at least one *rpoB* mutant melting peak, the result was designated heteroresistant (Fig. 2).

### FluoroType MTBDR

#### DNA extraction

MGIT960 growth culture (500 μL) was treated with 167 µL of inactivation reagent (room temperature, 30 min), and DNA was extracted using the GXT96 × 2 Extraction Kit VER1.0 (Bruker-Hain Lifescience, Nehren, Germany) with the GenoXtract fleXT instrument (Bruker–Hain Lifescience, Nehren, Germany), as per the manufacturer’s instructions (15, 16). With each extraction, saline buffer and un-inoculated MGIT960 (supplemented with PANTA) were included as a negative control, alongside the provided positive control.

#### PCR

Extracted DNA was amplified using the FluoroCycler XT (Bruker–Hain Lifescience, Nehren, Germany) and analyzed with the controls using FluoroSoftware XT-IVD (version 1.0.1.5.5.75; Bruker–Hain Lifescience, Nehren, Germany).

### Single molecule-overlapping reads (SMOR)

#### DNA extraction

Briefly, 100 µL of growth from the MGIT960 tube was heated at 100°C for 30 min, as previously described (17).

#### Sequencing

SMOR testing was conducted at the Translational Genomics Research Institute (T-Gen, Arizona, USA), where primers were used to amplify *inhA*, *katG*, and *rpoB* resistance-determining regions as described (Table S1) (11). A second PCR added adapters using a previously published universal tail method. Samples were pooled and sequenced on an Illumina MiSeq (V3, 600 bp paired-end chemistry). Multiple no-template controls were used as quality control to ensure the integrity of results.

#### Bioinformatics

The Amplicon Sequencing Analysis Pipeline (version 1.9; ASAP) was used (11, 12), which requires overlapping forward and reverse reads to agree, and uses read counts to report variant frequency. Resistance calls were classified by ASAP into predefined categories based on the percentage of reads with a known resistance-associated variant (RAV) as “microheteroresistance” (0.1%–<5%), “macroheteroresistant” (5%–95%) or “full resistant” (>95%) (8). SMOR requires at least 10 paired reads at a locus to make a call. In this case, to call to 0.1%, 10,000 paired reads were required for reporting. When multiple RAVs were detected in a single amplicon, ASAP was used to determine whether they were on the same read as previously described (18) and thus likely originate from a single population (haplotype identification).

### Statistical analysis and definitions

The 2 × 2 tables were used to calculate sensitivity and specificity with 95% confidence intervals (CIs, exact binomial method), and SMOR results were used as a reference standard for rifampicin and isoniazid. The prtesti command (STATA 18, StataCorp) was used for comparisons between proportions. Results were classified as successful if a test yielded a definitive resistant or susceptible result. Outcomes where MTBC was not detected or results were uninterpretable were classified as unsuccessful.

## RESULTS

### Frequency of discordant rifampicin results

Between 1 April 2021 and 30 September 2022, 1,623 patients with Ultra rifampicin resistance were identified. MTBDR*plus* was performed on 91% (1483/1623) of these samples, with 91% (1347/1483) yielding determinate results for rifampicin susceptibility. Of these, 8% (109/1347) were MTBDR*plus* rifampicin-susceptible, and hence discordant with Ultra (Fig. 1).

### Relationship between Ultra and SMOR rifampicin results

#### RAV frequency

Of 92% (100/109) Ultra-MTBDR*plus* discordant isolates available for SMOR, 97% (97/100) generated a successful result; 39% (38/97) were classified as fully resistant, 19% (18/97) macroheteroresistant, 21% (20/97) microheteroresistant, and the remainder 21% (20/97) had no resistance-associated reads. Therefore, the positive predictive value (PPV) of Ultra rifampicin resistance for true rifampicin resistance (as defined by a SMOR reference standard) was 79% (77/97), with 21% (20/97) of Ultra results correspondingly being false positive for rifampicin resistance. Lower Ultra-detected load (higher C_Tmin_) was positively associated with false-positive results (median [IQR] C_Tmin_ 29 [28–31] vs 19 [18–25] in true positives; *P* = 0.0001]. Specifically, in Ultra results with a “very low” semi-quantitation category, 46% (11/24) had false resistance compared with 12% (9/73, *P* = 0.0004) in those with a higher semi-quantitation category (when restricted to those with Ultra heteroresistant patterns, these were 75% [6/8] vs 21% [6/29]; *P* = 0.0037) (Table 1).

**TABLE 1:** Ultra parameters among true- and false-Ultra rifampicin-resistant results in people who were MTBDR*plus*-susceptible, using SMOR on DNA from isolates as a reference standard^*a*^

	True rifampicin-resistant (*n* = 77)	False rifampicin-resistant (*n* = 20)
C_Tmin_	19 (18–25)	29 (27–31); *P* = 0.0001
Semi-quantitation category		
High	40 (31/77)	5 (1/20); *P* = 0.0028
Medium	31 (24/77)	15 (3/20); *P* = 0.1506
Low	12 (9/77)	25 (5/20); *P* = 0.1312
Very low	17 (13/77)	55 (11/20); *P* = 0.0004
Specific probes with mutation label		
rpoB1 Mut	47 (32/68)	41 (7/17); *P* = 0.6633
rpoB2 Mut	13 (9/68)	18 (3/17); *P* = 0.6403
rpoB3 Mut	4 (3/68)	12 (2/17); *P* = 0.2491
rpoB4 Mut A	13 (9/68)	12 (2/17); *P* = 0.8716
rpoB4 Mut B	29 (2/68)	6 (1/17); *P* = 0.5567
More than one MUT probe	19 (13/68)	12 (2/17); *P* = 0.4769
Ultra heteroresistance pattern		
Heteroresistance pattern	32 (25/77)	60 (12/20); *P* = 0.0239

^*a*^ Ultra heteroresistance calls and lower detected bacillary load are more frequent among false-resistant results. Data are median (IQR) or % (n/N). C_Tmin_, cycle threshold minimum; HR, heteroresistance; MUT, mutation; Ultra, Xpert MTB/RIF Ultra. *P*-values are for within row comparisons across columns.

### Heteroresistance

Thirty-nine percent (38/97) of people had SMOR-detected heteroresistance (Table S2), and 38% (37/97) of Ultra results exhibited heteroresistant probe patterns. Among these Ultra results, 67% (25/37) had SMOR-detected resistance with two classified as resistant, 11 as macroheteroresistant, and 12 as microheteroresistance; 12 were classified as susceptible by SMOR. Of the 60 Ultra results without heteroresistant patterns, 87% (52/60) had SMOR-detected resistance, with 37 classified as resistant, seven as macroheteroresistant, and eight as microheteroresistant; eight were classified as susceptible by SMOR. SMOR-detected heteroresistance was more common in Ultra-detected heteroresistance isolates compared with those without Ultra-detected heteroresistance (62% [23/37] vs 25% [15/60]; *P* = 0.0003). Ultra heteroresistance patterns therefore had 61% (23/38) sensitivity and 95% (37/39) specificity for SMOR heteroresistance. Finally, Ultra heteroresistance was more likely than Ultra non-heteroresistance resistance results to be false positive for rifampicin resistance (PPV, 68% [25/37] vs 87% [52/60]; *P* = 0.021]).

#### Haplotyping

Isolates from 21% (16/77) people had two or more *rpo*B mutations detected by SMOR. Nineteen percent (3/16) had all mutant calls on the same read, and the remaining 79% (13/16) had mutations on separate reads, suggesting they were in separate strain subpopulations.

### Isoniazid susceptibility

Thirty-eight of 99 (38%) samples demonstrated isoniazid resistance-associated mutations by SMOR (22 resistant, seven macroheteroresistant, nine microheteroresistance; 61 susceptible); 55% (21/38) had *kat*G and 45% (17/38) *inh*A mutations by SMOR. Sensitivity and specificity for isoniazid resistance by MTBDR*plus* were 53% (20/38) and 98% (60/61), respectively (Table S3). Among the isolates that were false MTBDR*plus* isoniazid-susceptible, 67% (12/18) had heteroresistance (eight microheteroresistance). Heteroresistance was less frequent in MTBDR*plus* isoniazid true positives, with 20% (4/20) being heteroresistant (three macroheteroresistance). Notably, of the 77 isolates that were SMOR rifampicin-resistant, 56% (43/77) were isoniazid-susceptible (rifampicin mono-resistant).

### FluoroType MTBDR

### Rifampicin

From the usable rifampicin SMOR results, 91% (88/97) also had successful FT-MTBDR results, with 55% (48/88) resistant (Ultra-concordant) and 45% (40/88) susceptible (MTBDR*plus*-concordant). FT-MTBDR sensitivity and specificity for rifampicin resistance were 69% (47/68) and 95% (19/20), respectively (Table S3). Among FT-MTBDR rifampicin-susceptible isolates, 53% (21/40) were rifampicin-resistant via SMOR. Of these, 100% (21/21) had heteroresistance (19 microheteroresistant), and in all 100% (47/47), SMOR rifampicin-resistant isolates without heteroresistance were detected correctly by FT-MTBDR.

### Isoniazid

From the 98 people with successful isoniazid FT-MTBDR and SMOR results, 72% (71/98) were FT-MTBDR susceptible, and 28% (27/98) FT-MTBDR were resistant. FT-MTBDR sensitivity and specificity for isoniazid resistance were 71% (27/38) and 100% (60/60); respectively (Table S3). Among FT-MTBDR isoniazid-susceptible isolates, 15% (11/71) were isoniazid-resistant by SMOR. All of these, 100% (11/11) had heteroresistance (eight microheteroresistant), and 100% (22/22) SMOR isoniazid-resistant without heteroresistance were detected correctly by FT-MTBDR. Among people with SMOR heteroresistance, FT-MTBDR correctly detected resistance in 31% (5/16).

#### Compared with MTBDRplus for isoniazid resistance

99 people had successful MTBDR*plus* and FT-MTBDR results, 90% of which were concordant (19 resistant, 70 susceptible) and 10 discordant (eight FT-MTBDR resistant and MTBDR*plus* susceptible, two FT-MTBDR susceptible and MTBDR*plus* resistance; SMOR supported the FT-MTBDR result in 90% [9/10] people). The sensitivity of FT-MTBDR for isoniazid resistance was better than MTBDRp*lus* (53% [20/38] vs 71% [27/38]; *P* = 0.0983), whereas specificity remained similar (98% [60/61] vs 100% [60/60]; *P* = 0.3193) (Table S3).

## DISCUSSION

To our knowledge, this study is the first to describe rifampicin susceptibility discordance between the WHO-recommended rapid molecular tests Ultra and MTBDR*plus*. Our key findings are (i) most discordance (79%) was from MTBDR*plus* not detecting rifampicin resistance and (ii) 69% of these MTBDR*plus*-susceptible were detected as FT-MTBDR resistant, indicating that FT-MTBDR has higher sensitivity than MTBDR*plus.* However, (iii) a substantial proportion with sequencing-detected resistance (31%; all of which were heteroresistant) were missed by FT-MTBDR, and in people with heteroresistance, multiple resistant strains were often present. Furthermore, (iv) although the Ultra heteroresistance probe pattern was associated with heteroresistance, this pattern had suboptimal sensitivity and specificity for heteroresistance and was itself associated with Ultra false-resistant calls (as was lower mycobacterial load). Finally, (v) more than half of the isolates were rifampicin mono-resistant, supporting the need for isoniazid DST. These data have implications for laboratory DST algorithms, especially resolution of discordant results by different molecular methods.

Most rifampicin resistance discordance arose from MTBDR*plus* not detecting RAVs, rather than Ultra falsely detecting RAVs. This might be because MTBDR*plus* interpretation is subjective even with the semi-automated GenoScan and requires human reporting. In contrast, FT-MTBDR reporting is fully automated. While FT-MTBDR identified most resistance missed by MTBDR*plus*, approximately half of the isolates FT-MTBDR detected as rifampicin-susceptible had sequencing-detected resistance. This contrasts with other studies that have reported FT-MTBDR sensitivities approaching 100% (2); however, these were done in Ultra rifampicin-resistant people (without specifically selecting the discordant MTBDR*plus*-susceptible subset).

Heteroresistance, which we show to be a cause of Ultra-MTBDR*plus* discordance was, about a third of the time, missed by FT-MTBDR. However, as these people were MTBDR*plus*-susceptible, FT-MTBDR is still substantially better at detecting resistance than the previous generation technology. Heteroresistant *rpo*B mutations, including L511P, D516Y, and S531L, which were undetected by MTBDR*plus* but identified by SMOR, highlight the need for advanced tools to improve resistance detection and the potential inclusion of these regions in new tests for resistance. Interestingly, within people with sequencing-detected heteroresistance, there was seldom one resistant strain implicated, which is unexpected, given that these are not samples taken from people on treatment, and sequencing was done after culture, which can result in loss of minority variants (8). Possible causes of this diversity include multiple exposures to rifampicin-resistant MTBC or substantial intra-host evolution.

Certain probe patterns reported by Ultra have been proposed to be useful for diagnosing heteroresistance (10), which may be clinically useful if first-line drugs could be included in the regimen to rapidly reduce bacterial load of the drug-susceptible subpopulation (19, 20). However, in our study, although this Ultra probe pattern was indeed associated with heteroresistance, it did not translate into high sensitivity and specificity for heteroresistance. While FT-MTBDR does not currently offer a heteroresistance readout, this feature could be incorporated into its software to potentially inform treatment. Finally, this Ultra hetereoresistance pattern (as well as that from FT-MTBDR) was also associated with Ultra false-resistant calls, as was the Ultra “very low” semi-quantitation category. This category is a recognized risk factor for Xpert false resistance (6), for which repeat testing is recommended. Our data therefore suggest that samples with an Ultra heteroresistance pattern and or “very low” bacterial load should be considered at increased risk for false resistance. The utility of repeat testing in such samples warrants further evaluation.

Our findings emphasize the importance of not assuming rifampicin resistance equates to isoniazid resistance, particularly in cases of discordant Ultra-MTBDR*plus* results. Previous studies have demonstrated that 19% (21) and 21% (22) of Xpert rifampicin-resistant cases are isoniazid-susceptible by MTBDR*plus*. Our data therefore support the scale-up of upfront routine isoniazid DST to avoid the inappropriate exclusion of isoniazid from regimens.

Our study has strengths and limitations. Ultra was done on specimens, while MTBDR*plus*, FT-MTBDR, and SMOR were done on isolates. While this may be representative of some programmatic algorithms, changes in subpopulation structures due to culture bias could create discordance. Furthermore, phenotypic susceptibility testing was not possible as isolates were not stored to preserve viability. However, SMOR has high sensitivity and specificity for phenotypic (and sub-phenotypic) resistance (7). Another consideration is that our study was designed to investigate Ultra-resistant, MTBDR*plus-*susceptible discordance, rather than to assess Ultra rifampicin-resistant calls in all comers (which others have done for Ultra’s predecessor Xpert) (6). In other words, our findings should be interpreted within the context of samples pre-selected because they were Ultra-MTBDR*plus* discordant (such discordant samples are likely not representative of typical RAVs in our setting).

In conclusion, patients with Ultra rifampicin-resistant MTBC that were susceptible by MTBDR*plus* are predominantly truly rifampicin resistant unless Ultra produced a heteroresistant probe pattern or “very low” semi-quantitation category (both associated with false Ultra rifampicin results). Isoniazid, for which susceptibility testing should be done, likely remains useful in people with this Ultra-MTBDR*plus* discordance.

## Data Availability

Sequence data are available in NCBI under BioProject number PRJNA1217463. Study data can be accessed on request from the corresponding author without restriction.

## SUPPLEMENTAL MATERIAL

Supplemental tables aac.01671-24-s0001.docx Tables S1 to S3.

ASM does not own the copyrights to Supplemental Material that may be linked to, or accessed through, an article. The authors have granted ASM a non-exclusive, world-wide license to publish the Supplemental Material files. Please contact the corresponding author directly for reuse.
