# Supplementary material for: Xpert MTB/RIF Ultra-resistant and MTBDRplus-susceptible rifampicin results in people with tuberculosis: utility of FluoroType MTBDR and deep sequencing
Source: Antimicrob Agents Chemother. 2025 Feb 7;69(3):e01671-24. doi: 10.1128/aac.01671-24 (PMC11881552; doi:10.1128/aac.01671-24)
Supplement: Supplemental tables — Tables S1 to S3. [file aac.01671-24-s0001.docx]

**Supplementary Table of Contents:**

Supplementary Table 1. *Mycobacterium tuberculosis* complex specific primer with universal tail sequences……………………………………………………………………………………………….2

Supplementary Table 2. List of rpoB mutations not detected by MTBDRplus but detected by SMOR as heteroresistant.………………………………………………………………………………………….3

Supplementary Table 3. Sensitivity and specificity of FT-MTBDR and MTBDR*plus* for rifampicin or isoniazid resistance..……………………………………………………………..…..……….…………5

**Table 1.** *Mycobacterium tuberculosis* complex specific primer with universal tail sequences. All oligos are with standard de-salting. The universal tail sequences are highlighted in red and bold with the forward primer sequence differing from the reverse primer sequence. For *rpoB*, A 262-base pair segment, spanning positions 761,021 to 761,283 on the reference genome AL123456.3 was analyzed, encompassing the rifampicin resistance-determining region (RRDR) and extending beyond the canonical 81-base pair segment.

| **Forward Primer** | **Sequence** (5`-3`) |
| --- | --- |
| rpoBv2fUT1 | **ACCCAACTGAATGGAGC**CGATCACACCGCAGACGTT |
| katGv2fUT1 | **ACCCAACTGAATGGAGC**CCATGAACGACGTCGAAACAG |
| inhAv2fUT1 | **ACCCAACTGAATGGAGC**CCTCGCTGCCCAGAAAGG |
| **Reverse Primer** |  |
| rpoBv2rUT2 | **ACGCACTTGACTTGTCTTC**GTTTCGATCGGGCACATCC |
| katGv2rUT2 | **ACGCACTTGACTTGTCTTC**GCTCTTCGTCAGCTCCCACTC |
| inhAv2rUT2 | **ACGCACTTGACTTGTCTTC**GTCACATTCGACGCCAAACAG |

**Table 2.** List of *rpo*B mutations not detected by MTBDR*plus* but detected by SMOR as heteroresistant.

| **Study numbers** |  | Rifampicin DST results | | | | *rpo*B RAVs detected by SMOR |
| --- | --- | --- | --- | --- | --- | --- |
|  | **Semi quantification** | **MTBDR*plus*** | **Ultra** | **FT-MTBDR** | **SMOR** |  |
| DIS001 | Very Low | Susceptible | Resistant | Unsuccessful | MacroHR | L511P |
| DIS002 | Medium | Susceptible | Resistant | Resistant | MacroHR | L511P, D516Y |
| DIS003 | High | Susceptible | Resistant | Susceptible | MicroHR | H526Y |
| DIS004 | High | Susceptible | Resistant | Resistant | MacroHR | L511P |
| DIS005 | Very Low | Susceptible | Resistant | Susceptible | MicroHR | L511P |
| DIS007 | Low | Susceptible | Resistant | Susceptible | MicroHR | S531L |
| DIS008 | Medium | Susceptible | Resistant | Susceptible | MicroHR | S531L, D516A |
| DIS009 | High | Susceptible | Resistant | Susceptible | MicroHR | S531L |
| DIS010 | High | Susceptible | Resistant | Susceptible | MicroHR | S531L |
| DIS011 | Medium | Susceptible | Resistant | Susceptible | MicroHR | S531L |
| DIS012 | High | Susceptible | Resistant | Susceptible | MicroHR | S531L, D516V |
| DIS014 | High | Susceptible | Resistant | Susceptible | MicroHR | S531L |
| DIS015 | High | Susceptible | Resistant | Susceptible | MacroHR | S531L |
| DIS016 | High | Susceptible | Resistant | Susceptible | MicroHR | S531L |
| DIS028 | High | Susceptible | Resistant | Susceptible | MicroHR | S531L, L511P |
| DIS037 | High | Susceptible | Resistant | Resistant | MacroHR | D516Y, S531L |
| DIS042 | High | Susceptible | Resistant | Unsuccessful | MacroHR | L511P, H526L |
| DIS046 | Medium | Susceptible | Resistant | Susceptible | MicroHR | D516Y, L511P, S531L |
| DIS052 | Medium | Susceptible | Resistant | Resistant | MacroHR | S531L |
| DIS054 | High | Susceptible | Resistant | Unsuccessful | MacroHR | D516Y, H526D |
| DIS057 | Low | Susceptible | Resistant | Unsuccessful | MacroHR | D516G, S531L |
| DIS058 | Medium | Susceptible | Resistant | Susceptible | MicroHR | L511P, D516Y |
| DIS066 | Low | Susceptible | Resistant | Resistant | MacroHR | L511P, D516G |
| DIS075 | Medium | Susceptible | Resistant | Resistant | MacroHR | D516Y, S531L |
| DIS076 | High | Susceptible | Resistant | Unsuccessful | MacroHR | D516Y |
| DIS077 | Medium | Susceptible | Resistant | Resistant | MacroHR | D516Y |
| DIS083 | Very Low | Susceptible | Resistant | Resistant | MicroHR | D516Y |
| DIS085 | High | Susceptible | Resistant | Unsuccessful | MacroHR | L533P |
| DIS089 | High | Susceptible | Resistant | Susceptible | MicroHR | L511P |
| DIS093 | Medium | Susceptible | Resistant | Unsuccessful | MacroHR | Q513K, H526D |
| DIS095 | High | Susceptible | Resistant | Susceptible | MicroHR | H526R, S522L, S531L |
| DIS097 | High | Susceptible | Resistant | Unsuccessful | MacroHR | L511P |
| DIS098 | High | Susceptible | Resistant | Unsuccessful | MacroHR | L511P |
| DIS099 | Medium | Susceptible | Resistant | Susceptible | MicroHR | L511P, L533P |
| DIS100 | High | Susceptible | Resistant | Susceptible | MicroHR | L511P, L533P |
| DIS101 | Very Low | Susceptible | Resistant | Susceptible | MicroHR | L511P |
| DIS105 | Very Low | Susceptible | Resistant | Susceptible | MacroHR | S531L |
| DIS109 | High | Susceptible | Resistant | Susceptible | MicroHR | S531L |

Abbreviations: FT-MTBDR, FluoroType MTBDR; MicroHR, microheteroresistance; MacroHR, macroheteroresistance; MTBDR*plus*, MTBDR*plus* line probe assay; RAV, resistance-associated variant; SMOR, single molecule-overlapping repeats; Ultra, Xpert MTB/RIF Ultra

**Table 3.** Sensitivity and specificity of FT-MTBDR and MTBDR*plus* for rifampicin or isoniazid resistance detection when done on isolates from Ultra rifampicin-resistant MTBDR*plus* rifampicin-susceptible people using SMOR on DNA from isolates as a reference standard. FT-MTBDR sensitivity for each drug exceeded that of MTBDR*plus*. MTBDR*plus* specificity was higher for rifampicin and was lower for isoniazid than that of FT-MTBDR. Data are % (95% CI; n/N).

|  | Sensitivity | Specificity |
| --- | --- | --- |
| FT-MTBDR: Rifampicin | 69 (57-80; 47/68) | 95 (75-100; 19/20) |
| FT-MTBDR: Isoniazid | 71 (54-85; 27/38) | 100 (94-100; 60/60) |
| MTBDR*plus*: Rifampicin | Non-applicable | 100 (83-100; 20/20) |
| MTBDR*plus*: Isoniazid | 53 (39-69; 20/38) | 98 (91-100; 60/61) |

Abbreviations: FT-MTBDR, FluoroType MTBDR; MTBDR*plus*, MTBDR*plus* line probe assay; SMOR, single molecule-overlapping repeats; Ultra, Xpert MTB/RIF Ultra
